# Supplementary material for: Splice-Junction-Based Mapping of Alternative Isoforms in the Human Proteome
Source: Cell Rep. Author manuscript; Available in PMC 2020 Jan 15. (PMC6961840; doi:10.1016/j.celrep.2019.11.026)
Supplement: 2 [file NIHMS1546469-supplement-2.zip › DF1/PXD010154/Liver/Liver_56_NA_AQLVIYAGPNTNSRK.pdf]

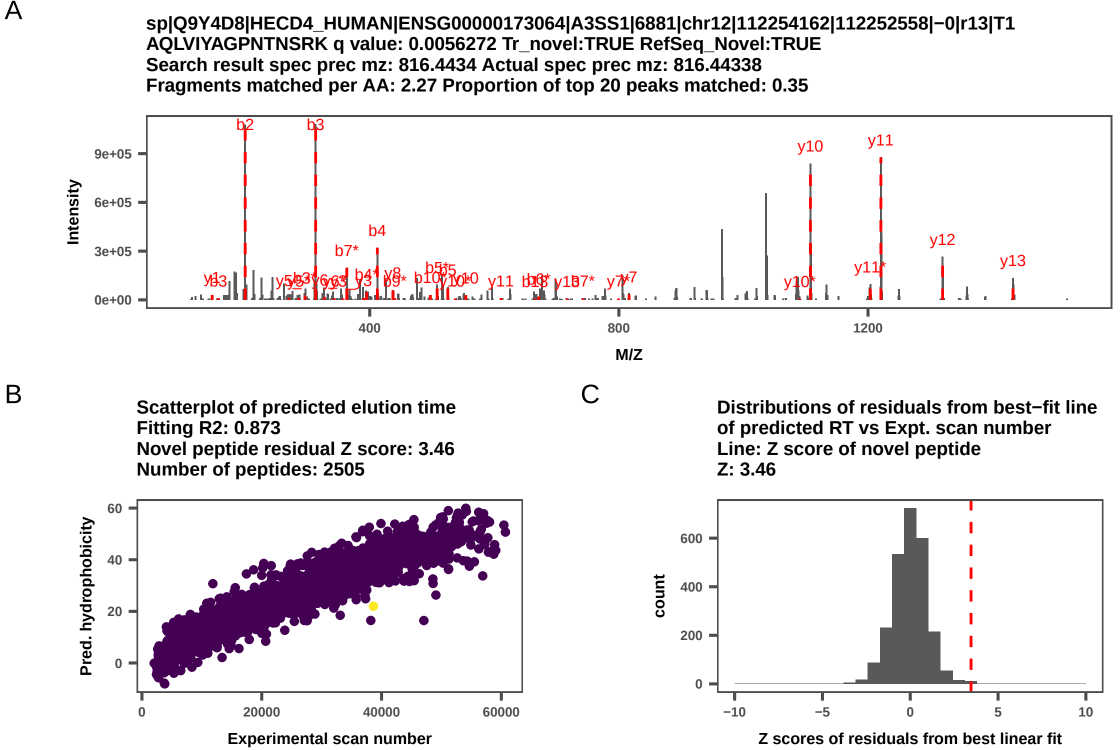
A
sp|Q9Y4D8|HECD4_HUMAN|ENSG00000173064|A3SS1|6881|chr12|112254162|112252558|−0|r13|T1
AQLVIYAGPNTNSRK q value: 0.0056272 Tr_novel:TRUE RefSeq_Novel:TRUE
Search result spec prec mz: 816.4434 Actual spec prec mz: 816.44338
Fragments matched per AA: 2.27 Proportion of top 20 peaks matched: 0.35
Intensity
9e+05
6e+05
3e+05
0e+00
b2
b3
b4
b7*
y10
y11
y12
y13
y11*
y10*
400
800
1200
M/Z
B
Scatterplot of predicted elution time
Fitting R2: 0.873
Novel peptide residual Z score: 3.46
Number of peptides: 2505
Pred. hydrophobicity
60
40
20
0
0
20000
40000
60000
Experimental scan number
C
Distributions of residuals from best−fit line
of predicted RT vs Expt. scan number
Line: Z score of novel peptide
Z: 3.46
count
600
400
200
0
−10
−5
0
5
10
Z scores of residuals from best linear fit
